# Study of Electron Ionization and Fragmentation of Non-hydrated and Hydrated Tetrahydrofuran Clusters

**DOI:** 10.1007/s13361-017-1634-y

**Published:** 2017-03-21

**Authors:** Michael Neustetter, Masoomeh Mahmoodi-Darian, Stephan Denifl

**Affiliations:** 1 Institut für Ionenphysik und Angewandte Physik and Center for Molecular Biosciences, Universität Innsbruck, Technikerstraße 25, 6020 Innsbruck, Austria; 2 Department of Physics, Karaj Branch, Islamic Azad University, Karaj, Iran

**Keywords:** Tetrahydrofuran, Hydration, DNA backbone, Cluster, Electron ionization, Radiation damage

## Abstract

Mass spectroscopic investigations on tetrahydrofuran (THF, C_4_H_8_O), a common model molecule of the DNA-backbone, have been carried out. We irradiated isolated THF and (hydrated) THF clusters with low energy electrons (electron energy ~70 eV) in order to study electron ionization and ionic fragmentation. For elucidation of fragmentation pathways, deuterated TDF (C_4_D_8_O) was investigated as well. One major observation is that the cluster environment shows overall a protective behavior on THF. However, also new fragmentation channels open in the cluster. In this context, we were able to solve a discrepancy in the literature about the fragment ion peak at mass 55 u in the electron ionization mass spectrum of THF. We ascribe this ion yield to the fragmentation of ionized THF clusters.

**Graphical Abstract:**
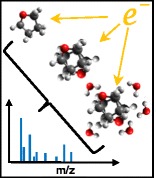
ᅟ

ᅟ

## Introduction

Whenever highly energetic radiation interacts with biological tissue, many secondary species, mainly low-energy electrons (LE﻿Es;typically <100 eV) are produced, which subsequently lose their energy by collisions [1]. The secondary electrons may cause damages in the repeated sugar-phosphate backbones and also in the four fundamental nucleic bases attached to the backbone by covalent bonds, ruptured through the process of dissociative electron attachment (DEA) [2, 3], electronic excitation of neutrals, and dissociative ionization. Sanche and co-workers [2, 3] have shown that low energy electrons interacting with plasmid DNA induce single-strand breaks (SSBs) and double-strand breaks (DSBs), thereby leading to cell damage. Due to these observations, numerous investigations concerning electron interactions with isolated molecules related to DNA have been performed within the last decade, e.g., [4–7]. As known, gas-phase studies constitute a powerful method for describing fundamental processes at the molecular level. However, for an isolated system, the molecular dynamics initiated by a collision cannot be affected by the surrounding medium, which may occur in the condensed phase. To draw conclusions on such environmental effects, experiments with clusters of DNA constituents are important to fill the gap between isolated molecules and condensed matter. Hereby, hydrated biomolecules are of interest due to the fact that the body consists of up to 70% water. In the case of electron attachment, experiments were recently done by Neustetter et al. [8]. For this purpose, they studied electron attachment to non-hydrated and hydrated pyrimidine clusters. Pyrimidine as a derivative of the nucleobases thymine, cytosine, and uracil is often used as a model molecule. Their observations showed that in comparison to the isolated molecules, clusters show an extraordinary effect of solvation, which means DNA and RNA bases should be protected overall from LEEs in radiated cells by the surrounding water. This behavior was also found by Kocisek et al. who very recently investigated electron attachment to hydrated uracil and thymine [9].

Avaldi and Huber [10] investigated the fragmentation of isolated uracil molecules as well as non-hydrated and hydrated uracil clusters induced by collisions with ^12^C^4+^ cations. They showed that overall the surrounding water has a protecting effect, reducing the damage and the yield of low mass fragments by one to two orders of magnitude for non-hydrated and hydrated uracil clusters, respectively. However, the cluster environment was also responsible for the opening of new fragmentation channels and formation of new species as a result of the altered bonding situation. Studies on electron ionization as well as multi-photon ionization of hydrated nucleobases are also available [11–14]. For example, Barc et al. [11] carried out multi-photon and electron ionization experiments with adenine-water clusters. Also, their measurements suggested a protective effect on the water surrounded nucleobase adenine.

In order to determine the most sensitive part of the DNA or RNA to electron-induced bond rupture, surface experiments with nucleic bases and phosphate-sugar groups were carried out (see, e.g., [15–17]). Those studies demonstrated that the sugar deoxyribose (2-deoxy-D-ribose) is the major target of electrons in producing strand breaks in DNA or RNA. These findings are in agreement with studies of Ptasinska et al. [18], who showed that the isolated deoxyribose molecule is extremely fragile in terms of electron interactions. Theoretical studies revealed a very efficient fragmentation of the deoxyribose [19] also in the interaction with protons.

Hence it is important to investigate the electron interaction of hydrated sugar-phosphate groups. In the present study, we investigate the ionization and fragmentation of tetrahydrofuran [THF, (CH_2_)_4_O] by electron ionization. THF is often regarded as the simplest molecular analog or prototype of deoxyribose, which is part of the backbone of DNA. Tetrahydrofuran is a cyclic ether with one oxygen atom and four (CH_2_) units in the five-membered ring. The ring is a constituent of deoxyribose in furanose form. Thus, a number of studies have been undertaken on electron collisions with THF, for example, the measurements of total cross-sections [20–24], differential elastic and inelastic scattering cross-sections [23–28], and electron ionization dynamics of THF [29–33].

Herein, we describe the fragmentation of positively charged non-hydrated and hydrated tetrahydrofuran clusters formed via electron ionization at electron energies of ~70 eV. We compare cation formation for the isolated molecule with that in clusters, which enables the investigation of the effects of the surroundings on the process induced in biological matter. Since THF and the tetramer water-cluster have the same nominal mass, fully deuterated THF [TDF, (CD_2_)_4_O] has been used too. In addition, the comparison of the spectra of THF and TDF resolves some inconsistencies between NIST data and mass spectra published in several papers, as will be described below.

## Experimental

**Figure 1:**
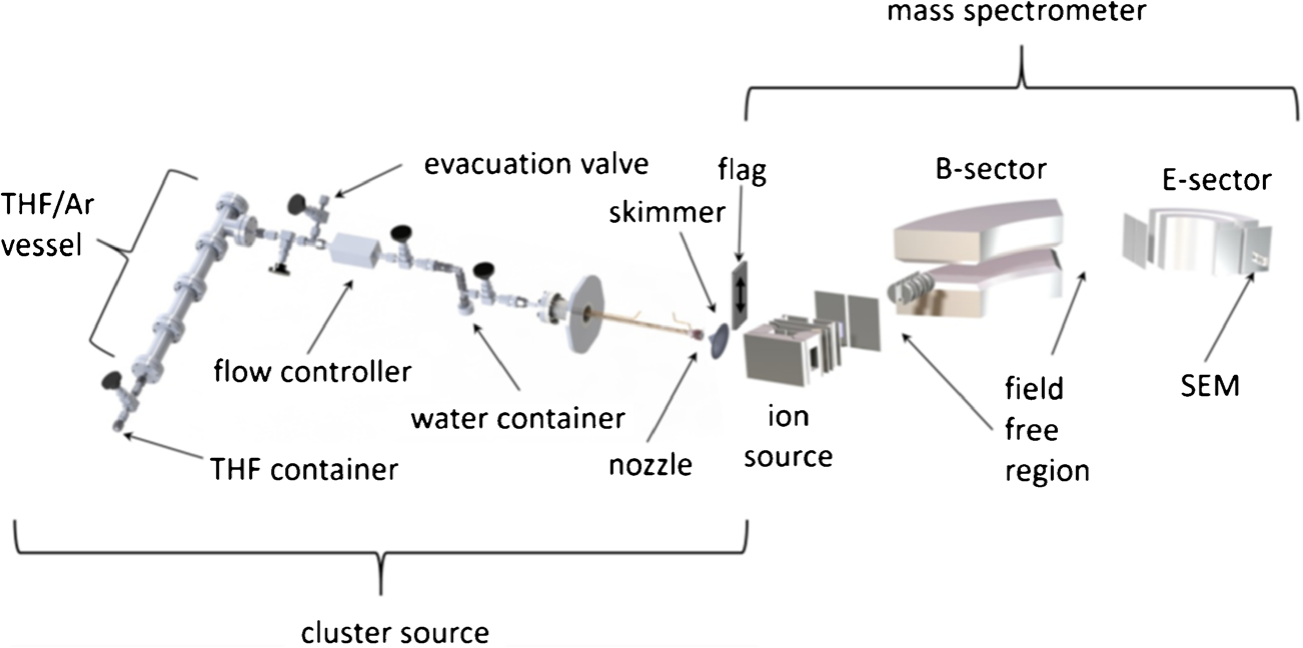
Experimental setup consisting of the homemade cluster source and the double-focusing sectorfield mass spectrometer. The most important parts are labeled accordingly

The experiments were performed by means of a homemade supersonic cluster beam source linked to a double-focusing sector field mass spectrometer. A scheme of the experimental setup is shown in Figure 1. The clusters were formed by supersonic expansion in a cluster beam source consisting of nozzle, water container, and sample reservoir. The sample as well as the water was located outside the vacuum chamber. This configuration enabled exchanging or refilling the components quickly without breaking the vacuum. Five different heating systems permitted heating the entire source, including the nozzle, the sample reservoir, and water container. Due to the high vapor pressure of tetrahydrofuran [143 mm Hg (20 °C)], [34], it was not necessary to heat the sample during the measurements.

However, to improve the clustering argon (Ar, 2–5 bars) was used as seeding gas. For the present measurements, the sample and the argon were mixed in a separate vessel. The water container was located right after the THF/Ar reservoir in such a way that the THF/Ar mixture had to pass the water vapor before the expansion. For the measurements without water, the container was kept in the system but dried. It was possible to heat the nozzle as well as the tube inside and outside the chamber separately. During the studies, the orifice and the tube inside the chamber were heated up to 30 °C to avoid condensation and clogging, respectively. The water container and the sample were kept at room temperature. The measurements were obtained with a 50 μm pin hole orifice distanced 10 mm in front of a skimmer from Beam Dynamics Inc., Jacksonville, USA, (1 mm in diameter), which separated the ion source chamber and the cluster chamber. By such configuration, the chambers were pumped differentially to keep the pressure in the ion source chamber as low as possible. Additionally, a cryostat was installed in the upper part of the cluster chamber acting as cold trap at a temperature of ~24 K. During the measurements, the pressure in the cluster source was kept at about 4–6 × 10^-4^ mbar, leading to a pressure of 1–4 × 10^-6^ mbar in the ion source. To keep the pressure constant, a flow controller was installed regulating the THF/Ar flow coming from the reservoir. A moveable flag (simple metal plate) was installed between skimmer and ion source (see Figure 1). This flag enabled distinguishing between ion signal generated from the cluster beam and ion yield formed by ionization of residual gas present in the ion source chamber, respectively [35]. The cluster ion yield was corrected for this background by subtracting the signals of the flag measurement. For ionization, a standard Nier-type ion source [36] was used. If not explicitly mentioned, an electron current of 10 μA and an electron energy of 70 eV were chosen to record the present data. The formed ions were accelerated towards the mass analyzer with an acceleration voltage of 6 kV. The utilized mass analyzer has already been described in detail (e.g., [37]). A double-focusing sector field mass spectrometer in reversed Nier-Johnson geometry (VG-ZAB-2SE) was used to identify the produced ions. After a field-free region, the ions had to pass a magnetic field analyzing their momentum. Subsequently they again entered a field-free region followed by an electric field analyzing their energy. With this configuration, we achieved a mass-resolution of about 1500 [full width at half maximum (FWHM)]. A channeltron-type electron multiplier was used to detect the ion signal. The investigated samples were purchased from Sigma Aldrich, Vienna, Austria; according to the datasheets a purity of ≥ 99.9% in case of THF and 99.5% deuteration in case of the deuterated tetrahydrofuran (TDF) were provided.

## Results and Discussion

### Below the Monomer (Non-Hydrated)

**Figure 2:**
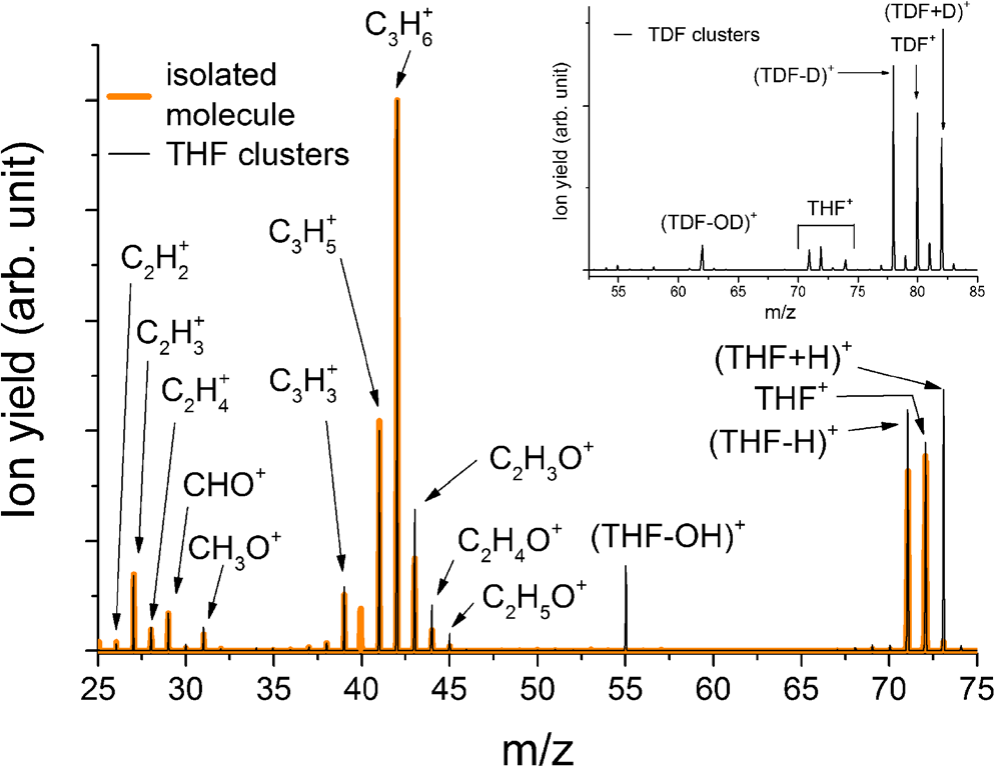
Obtained mass spectrum of THF in the mass region below the monomer. The signal derived from the cluster beam (Ar pressure: 5 bars) is compared with the isolated molecule. The inset shows a section of the mass spectrum of clustered TDF (Ar pressure: 2 bars). The comparison of the spectra reveals a OH/OD loss (55 u/62 u), which only happens at clustering conditions. The TDF mass scan (inset) further indicates a weak contamination of the T﻿DF sample with THF. The ion yield at mass 40 u in the THF cluster spectrum was not measured in order to prevent saturation of the detector

In Figure 2, the ion signal obtained from the THF cluster beam is plotted in the range below the monomer (25 to 75 u). For comparison, the electron ionization mass spectrum of isolated THF measured with the same setup is also included in the Figure. This mass spectrum was obtained by introducing THF as stagnant gas into the ion source chamber. For a better comparison, the spectra are scaled at mass 42 u, the most intense fragment ion peak corresponding to C_3_H_6_^+^. Comparing the cluster signal with the isolated molecule, one can clearly see that the fragmentation pattern in the mass region from 26 to 44 u does not change significantly. We ascribe small differences below ~10% (as e.g., observed in the case of C_3_H_5_^+^) to experimental effects, such as slightly different extraction efficiencies for ions formed from the beam and from stagnant gas. Nevertheless, the yields of fragment ions with the composition C_2_H_y_O^+^ (y = 3–5) seem to be enhanced relative to C_3_H_6_^+^. The parent cation [(THF)^+^ at 72 u] and the dehydrogenated tetrahydrofuran ion [(THF-H)^+^ at 71 u] are also present in both spectra. The latter is slightly enhanced in the cluster spectrum relative to C_3_H_6_^+^. (THF-H)^+^ is formed via C–H bond cleavage and is also likely an intermediate product in the sequential decay of the parent ions formed with sufficient excess energy. The same can be expected for the ions C_2_H_y_O^+^ (y = 3–5). In the cluster environment, the distribution of this excess energy within the cluster may be responsible to freeze the decay process, which leads to the enhanced formation of these fragment ions. It should also be pointed out that the clusters formed in the expansion have a reduced temperature compared with the isolated molecules, which are ionized at ambient temperatures. Temperature effects in electron ionization were reported for few molecules [38], which resulted from the different vibrational energies of the molecular ions with temperature. A different temperature may also lead to a change of dominant structural conformations. In [39], two almost iso-energetic minima for the isolated THF molecule were reported: the twisted conformer (with two energetically equal forms) and the envelope form. In contrast, for small clusters of THF, a preference for the twisted conformer was observed [39].

Furthermore, in Figure 2 two novel peaks generated by the fragmentation of THF clusters can be observed, namely at mass 55 and 73 u. Based on the natural isotope abundances, the ratio between the THF isotopes with 73 and 72 u should be about 4.5% [40]. The ratio is larger for clusters (dependent on the expansion conditions), which indicates the presence of the protonated molecule. In the THF spectrum shown, the protonated monomer (THF + H)^+^ is more abundant than (THF)^+^. The observation of protonated THF was already repored by Sharma et al. who investigated multiple photon ionization of THF clusters [41]. The formation of (THF + H)^+^ by the ionization of clusters is due to the high proton affinity of THF, which is about 8.5 eV [42].

A literature survey reveals inconstencies concerning the peak at 55 u because the peak was not reported in each electron ionization study with isolated THF. Fuss et al. [20], Dampc et al. [31], and Ren et al. [43] were able to detect it, whereas Gallegos and Kiser [32], Rudeck et al. [44], and Collin and Conde-Caprace [33], a well as the NIST database [45] do not mention it. Dampc et al. [31] assigned this peak to impurities of the sample since the intensity decreased with time of the measurements. In the mass spectrum presented by Ren et al. [43], the peak is clearly visible, but they had no clue about its origin. Fuss et al. [20] obtained a peak around 55 u, which was more than two times higher than the monomer, but the mass resolution was too low to determine the exact mass.

As mentioned by Dampc et al. [31], the peak at 55 u could result from an impurity. In [46] the reaction of THF with O_2_ was reported leading to the formation of the butanal 3-hydroxy molecule, which can form a fragment cation with the mass 55 u upon ionization [45]. We considered this hypothesis as well because of lack of information on possible impurities. However, the sample purity stated by the supplier (Sigma Aldrich) is 99.9% (i.e., the peak at mass 55 u at clustering conditions is too intense to be caused by an impurity). To rule out a possible reaction with oxygen that is present as residual air when filling the sample, we filled the THF into the sample container at two different conditions. Once under protective atmosphere created by Ar and once exposed to air. If a reaction of THF with O_2_ would take place, the 55 u peak would be enhanced compared with the parent THF peak, when exposed to air. Since this was not the case, the formation via a reaction channel with O_2_ can be excluded. Another hypothesis would include the contamination of the ion source by e.g., pump oil or previous samples. As mentioned in the Experimental section, we were able to block our cluster beam and thus distinguish between signal derived from the cluster beam and residual gas in the chamber. This allows us to substract the background, which can be assumed as constant and thus for both conditions (flag versus cluster) the same. Hence, Figure 2 clearly shows that the peak at mass 55 u results from a fragmentation channel of THF clusters. A closer look at the experimental setup used by Ren et al. [43] or Fuss et al. [20], respectively, reveals that it was likely possible with their conditions to create clusters.

To our knowledge, explicit studies of cationic non-hydrated THF clusters are scarce so far. Two photon-ionization studies were published by Sharma et al. [41, 47]. They used a pulsed nozzle system to generate the clusters via supersonic expansion. The ions were formed by multi-photon ionization with nanosecond laser pulses. They obtained mass spectra at three different wavelengths (355, 532, and 1064 nm). In contrast to our results, they also do not mention any peak around 55 u. Another contrast to the presently recorded electron ionization spectra is the major abundance of light fragment cations (e.g., C_2_^+^) observed in [41].

Our measurements show only one noteworthy peak in the mass range around 55 u. Fuss et al. [20] proposed two possible fragments with this mass, namely C_4_H_7_^+^ and C_3_H_3_O^+^. For a distinct identification, we repeated the measurement with deuterated tetrahydrofuran [(CD_2_)_4_O, TDF]. The resulting cluster spectrum is shown in the inset of Figure 2. Due to contamination of our cluster source, weak signal from THF is present between 70 and 75 u. However, the 55 u peak is not present anymore (the remaining weak ion yield can be ascribed to contaminations by THF, see inset of Figure 2), whereas a new peak appeared at mass 62 u, which again vanished by blocking the beam. This signal is due to C_4_D_7_^+^, which can be produced via the secession of OD from a TDF molecule (80 u). As a result, the 55 u peak in the case of THF corresponds to the loss of an OH molecule, forming C_4_H_7_^+^. To split-up OH/OD, two C–O bonds and one C–H/D bond have to be broken.

### Above the Monomer (Non-Hydrated)

**Figure 3:**
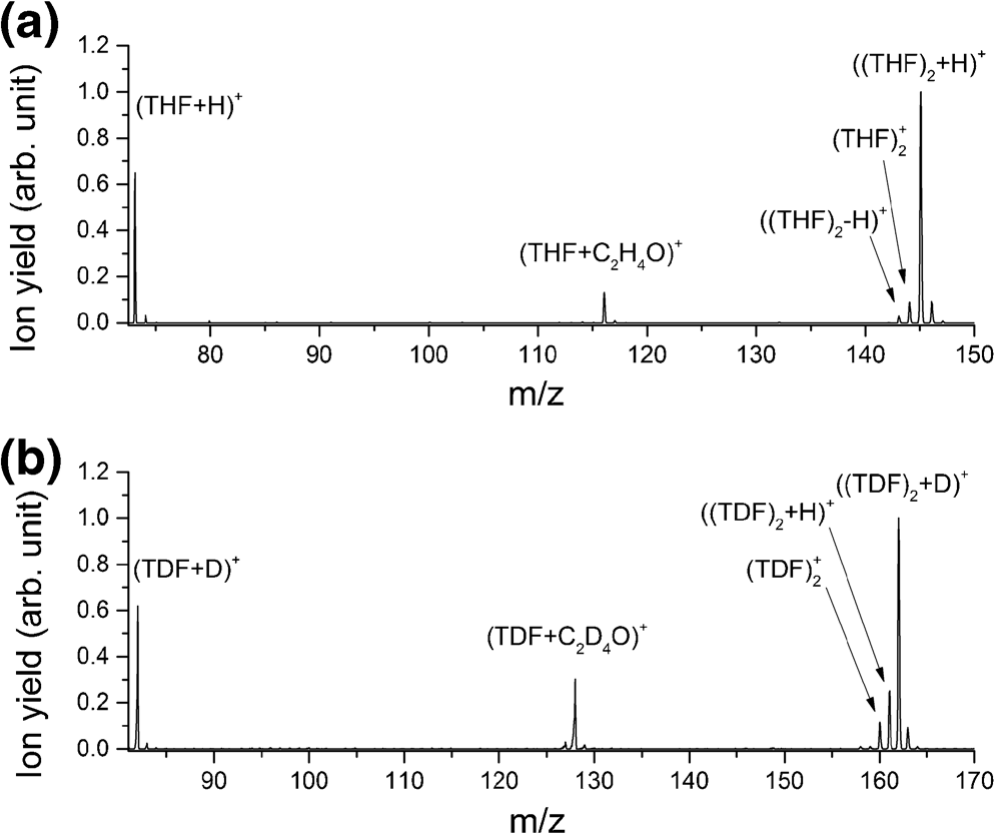
Obtained mass spectra of non-hydrated THF clusters (**a**) and TDF clusters (**b**) in the mass range between 73 and 150 u, and 81 to 170 u, respectively. The most abundant fragment ion between the protonated monomer and the dimer in both cases is caused by a C_2_H_4_/C_2_D_4_ loss

Figure 3 presents the resulting mass spectra of non-hydrated THF clusters (**a**) and TDF clusters (**b**) in the mass region between 73 and 150 u, and 81 and 170 u, respectively. In both cases, the protonated dimer [(THF)_2_ + H]^+^ and [(TDF)_2_ + D]^+^, respectively, are dominant compared with the dehydrogenated dimer species, and are used for scaling of the spectra. The same observation was seen by Sharma et al. [41]. Similar to the case of the protonated monomers, the protonated dimers are produced via fragmentation of larger neutral clusters, which have to be at least a trimer.

In both cases, deuterated (Figure 3b) as well as non-deuterated (Figure 3a), an intense peak between monomer and dimer is visible. The peaks are located at mass 128 and 116 u, respectively, which corresponds to (TDF + C_2_D_4_O)^+^ and (THF + C_2_H_4_O)^+^. The formation of those fragments is only possible via ring-cleavage and loss of two CD_2_/CH_2_ groups from a molecule of the cluster. Consequently, either two C–C bonds or one C–C bond in combination with a C–O bond have to be broken to lose C_2_H_4_/C_2_D_4_. Based on our measurements, we are not able to draw conclusions on the process in terms of which bonds are broken. To our knowledge, three different suggestions about the geometrical structure of (THF)_2_ exist [39, 48–50]. Hence, the structure cannot be used as indication for the origin.

Boese et al. [39] as well as Mizuno et al. [50] calculated a dimer-geometry where both molecules are stacked with the oxygen atoms in opposite directions. In contrast, Gargano and co-workers [48] calculated an almost T-like structure. This confirmed the simulations of Bowron et al. [49], which showed that the creation of larger clusters is done by T-like packing of the molecules. However, independent of the neutral precursor-cluster size, the energy necessary to break those intramolecular bonds is considerably higher than the strength of intermolecular bonds within the cluster. A similar observation (formation of molecular fragments attached to intact clusters) were recently made by Avaldi and co-workers [10]. They described this phenomenon as a nonstatistical process associated with a localized energy deposition followed by ultrafast dissociation before energy redistribution.

Both spectra of Figure 3 are scaled at the protonated dimer signals. Noticable are the differences in the relative ion yields of (THF + C_2_H_4_O)^+^ and (TDF + C_2_D_4_O)^+^, whereas the ratios of the protonated/deuteronated monomer species stays the same. This leads to the assumption that the protonated species (monomer and dimer) have the same origin, which is not identical with the origin of the fragments formed via C_2_H_4_/C_2_D_4_ secession.

Noteworthy is the intense peak of [(TDF)_2_ + H]^+^ at mass 161 u (Figure 3b). This signal could be explained by the fragmentation of mixed clusters (e.g., with water), which cannot be excluded. Nevertheless, the absence of other cationic signals, which should also be produced by ionization of such mixed aggregates, hints that the signal of [(TDF)_2_ + H]^+^ is not caused by mixed clusters. Another possibility is the exchange of a deuterium with a hydrogen. This was possible either by the interaction with residual humidity present in the system, a reaction with THF that was present from previous measurements, or the exposure to residual moisture in the course of the filling process of the sample container. With an intensity of about 25%, the exchange of one deuterium seems to be a favorable process.

The comparison of our results with the measurements obtained by Sharma et al. [47] reveals a huge difference. In contrast to us, they were not able to detect any fragments between the protonated monomer and the dimer. The absence of the (THF + C_2_H_4_O)^+^ can be explained by the different types of ionization techniques. As already mentioned in the previous section, they ionized their clusters via multi-photon interactions. As they described, it was very likely for their experiment to produce multiple ionization centers, which are not stable and thus decay. The instability (short lifetime) of doubly charged THF was already explained by Mayer et al. [51]. In this case the cluster dissociates and the energy necessary for the secession of two CH_2_ groups cannot be transferred quickly enough into the corresponding degrees of freedom.

### Hydrated Clusters

**Figure 4:**
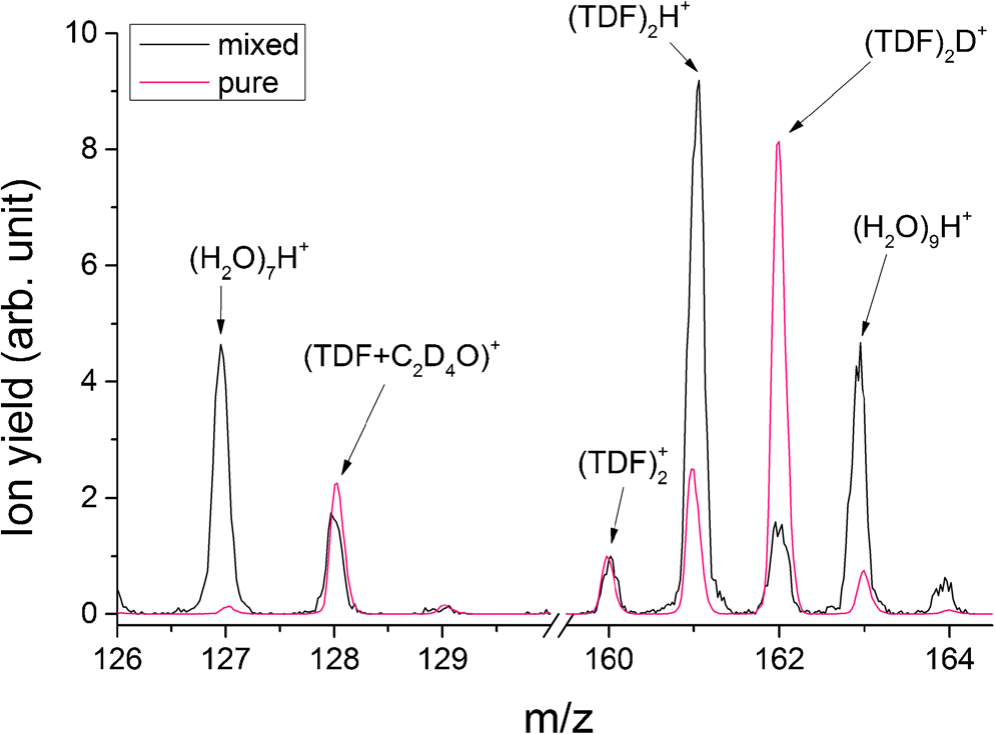
Comparison of non-hydrated (black) and hydrated (pink) TDF clusters in the mass range of 126–130 u, and 160–165 u. Beside the pure protonated water clusters [(H_2_O)_7_H^+^, (H_2_O)_9_H^+^], the increased signal of the protonated TDF dimer cation (TDF)_2_H^+^ is clearly visible when hydrated while the cation yield of (TDF)_2_D^+^ decreased. The fragment ion at 128 u corresponding to (TDF + C_2_D_4_O)^+^ is about 20% lower in the hydrated case. The spectra are scaled at mass 160 u, which corresponds to (TDF)_2_^+^

Based on the fact that THF and (H_2_O)_4_ have the same nominal mass, we are not able to distinguish between the mixed clusters (THF + (H_2_O)_n_)^+^ and the pure water clusters (H_2_O)_m_^+^, (m ≥ 4). Thus we investigated the hydration of TDF. The resulting spectra of the mixed expansion (TDF with H_2_O) revealed two different cluster series, namely pure water clusters and TDF-water mixtures. In both cases, the protonated species [(H_2_O)_i_H^+^ and (TDF)(H_2_O)_j_H^+^, respectively] are pronounced compared with the non-protonated species. The preferable formation of protonated water clusters due to electron ionization is already a well-known phenomenon [52]. As shown in Figure 4 (spectrum of hydrated TDF in comparison with non-hydrated TDF clusters), the signals of (TDF)(H_2_O)_j_H^+^ are in the hydrated case much more intense compared with the (TDF)(H_2_O)_j_D^+^. The former are not produced via a fragmentation channel of a TDF molecule but instead by ionization and subsequent fragmentation of the water. The transition of the most intense signal from (TDF)_2_D^+^ (non-hydrated TDF clusters) to (TDF)_2_H^+^ (hydrated TDF clusters) is an indication for a protective behavior of the water environment. Only the dissociation of the water molecule can lead to a hydrogen attachment to the TDF dimer, whereas a dissociation of the TDF molecule would lead to a deuteron attachment. The favorable destruction of the water molecules in the hydrated case was also seen by Kresin and co-workers [53] who investigated hydrated amino acids such as glycine and trypophan. Theoretical investigations on the structure of neutral hydrated THF molecules (up to four water molecules) were done by Vallejos et al. [54]. The THF molecule is both hydrophilic (on the oxygen side) and hydrophobic (on the CH_2_ groups) [54]. However, they presented that in the case of one water molecule, the hydrogen bond is created on the hydrophilic oxygen side. This changes with the presence of a second water molecule, forming an additional bond to a CH_2_ group. The same behavior was observed for three H_2_O molecules but changed when adding a fourth water molecule. In this case, the bond formation to a CH_2_ group is energetically unlikely to happen. Taking into account the geometry predicted by Vallejos et al. [54], the water complex (even for low amounts of hydration) is much bigger than the THF itself and thus it is plausible that the electron is preferably interacting with the water. This fits very well with our observation of preferential proton attachment compared with the attachment of a deuteron, and again supports the hypothesis of a protective behavior of the water surrounding.

Additionally in Figure 4, the region of the most abundant fragment peak formed via ring-cleavage is shown in the mass region between 126 and 128 u. Besides the pure protonated water cluster peak (H_2_O)_7_H^+^ at mass 127 u, the signal caused by the loss of two CD_2_ groups is still the highest peak in this mass range. Compared with the results for non-hydrated clusters, the intensity of this peak is in the hydrated case about 20% lower, which again reflects the protective behavior of the water environment.

## Conclusion

This work reports measurements of the cation mass spectra of isolated THF molecules and THF clusters (non-hydrated and hydrated) using a double-focusing sector field mass spectrometer. A number of well resolved mass peaks were detected and assigned to the corresponding ionic molecular fragments. The cation mass spectrum of the isolated THF molecule was compared with non-hydrated THF clusters, as well as the latter with hydrated clusters. The presence of the cluster matrix also leads to opening of new fragmentation channels like (THF-OH)^+^ appearing at mass 55 u. By the identification of this signal as a fragment ion formed by ionization of clusters, the inconsistency concerning mass 55 u in electron ionization mass spectra can also be removed.

Electron ionization of non-hydrated THF/TDF clusters showed, besides the efficient secession of H/D, one heavier fragment formed via ring-cleavage. The comparison of both mass spectra (from THF and TDF) allowed identifying this fragment ion as (THF + C_2_H_4_O)^+^/(TDF + C_2_D_4_O)^+^ formed by the loss of two CH_2_/CD_2_ groups. For hydrated clusters we observed a protective behavior of the surrounding water, which quenches dissociation of THF.
